# 3-(4-Fluoro­phenyl­sulfin­yl)-2,4,6,7-tetra­methyl-1-benzofuran

**DOI:** 10.1107/S1600536810005702

**Published:** 2010-02-17

**Authors:** Hong Dae Choi, Pil Ja Seo, Byeng Wha Son, Uk Lee

**Affiliations:** a Department of Chemistry, Dongeui University, San 24 Kaya-dong Busanjin-gu, Busan 614-714, Republic of Korea; b Department of Chemistry, Pukyong National University, 599-1 Daeyeon 3-dong, Nam-gu, Busan 608-737, Republic of Korea

## Abstract

In the title compound, C_18_H_17_FO_2_S, the 4-fluoro­phenyl ring is almost perpendicular to the benzofuran fragment [88.07 (5)°]. The crystal structure exhibits weak inter­molecular C—H⋯O hydrogen bonds and C—H⋯π inter­actions. The mol­ecules form pseudo-helices along the *a* axis.

## Related literature

For the crystal structures of similar 2-methyl-3-phenyl­sulfinyl-1-benzofuran derivatives, see: Choi *et al.* (2007 ▶, 2008*a* ▶,*b* ▶). For the biological activity of benzofuran compounds, see: Aslam *et al.* (2006 ▶); Galal *et al.* (2009 ▶); Khan *et al.* (2005 ▶). For natural products with benzofuran rings, see: Akgul & Anil (2003 ▶); Soekamto *et al.* (2003 ▶).
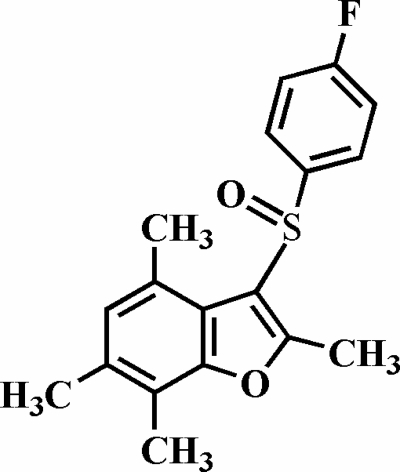


## Experimental

### 

#### Crystal data

C_18_H_17_FO_2_S
*M* *_r_* = 316.38
Orthorhombic, 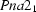

*a* = 12.0034 (5) Å
*b* = 19.7455 (7) Å
*c* = 6.4918 (3) Å
*V* = 1538.64 (11) Å^3^
*Z* = 4
Mo *K*α radiation
μ = 0.23 mm^−1^
*T* = 169 K
0.18 × 0.17 × 0.16 mm

#### Data collection

Bruker SMART APEXII CCD diffractometer
Absorption correction: multi-scan (*SADABS*; Bruker, 2009 ▶) *T*_min_ = 0.644, *T*_max_ = 0.746
8107 measured reflections
2280 independent reflections
2175 reflections with *I* > 2σ(*I*)
*R*_int_ = 0.024

#### Refinement

*R*[*F* ^2^ > 2σ(*F* ^2^)] = 0.032
*wR*(*F* ^2^) = 0.082
*S* = 1.06
2280 reflections
204 parameters
1 restraint
H-atom parameters constrained
Δρ_max_ = 0.25 e Å^−3^
Δρ_min_ = −0.28 e Å^−3^
Absolute structure: Flack (1983 ▶), 366 Friedel pairs
Flack parameter: 0.01 (12)

### 

Data collection: *APEX2* (Bruker, 2009 ▶); cell refinement: *SAINT* (Bruker, 2009 ▶); data reduction: *SAINT*; program(s) used to solve structure: *SHELXS97* (Sheldrick, 2008 ▶); program(s) used to refine structure: *SHELXL97* (Sheldrick, 2008 ▶); molecular graphics: *ORTEP-3* (Farrugia, 1997 ▶) and *DIAMOND* (Brandenburg, 1998 ▶); software used to prepare material for publication: *SHELXL97*.

## Supplementary Material

Crystal structure: contains datablocks I. DOI: 10.1107/S1600536810005702/gw2077sup1.cif

Structure factors: contains datablocks I. DOI: 10.1107/S1600536810005702/gw2077Isup2.hkl

Additional supplementary materials:  crystallographic information; 3D view; checkCIF report

## Figures and Tables

**Table 1:** Hydrogen-bond geometry (Å, °) *Cg* is the centroid of the C13–C18 ring.

*D*—H⋯*A*	*D*—H	H⋯*A*	*D*⋯*A*	*D*—H⋯*A*
C15—H15⋯O1^i^	0.95	2.59	3.529 (3)	169
C18—H18⋯O2^ii^	0.95	2.49	3.328 (2)	147
C11—H11*A*⋯*Cg*^ii^	0.98	2.66	3.553 (3)	152
C12—H12*A*⋯*Cg*^iii^	0.98	2.78	3.590 (3)	141

Symmetry codes: (i) 
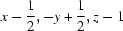
; (ii) 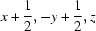; (iii) 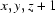.

**Fig. 1.**
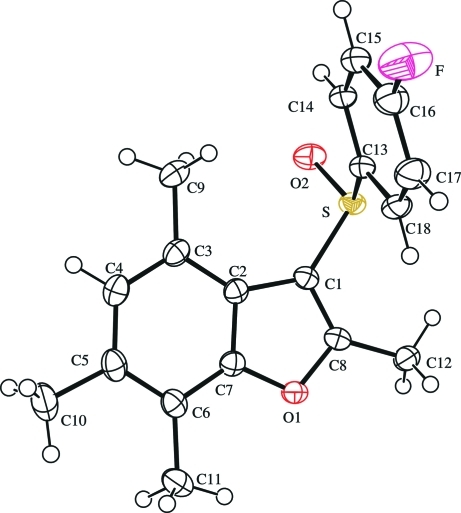
The molecular structure of the title compound with the atom numbering scheme. Displacement ellipsoids are drawn at the 50 % probability level. H atoms are presented as a small spheres of arbitrary radius.

**Fig. 2.**
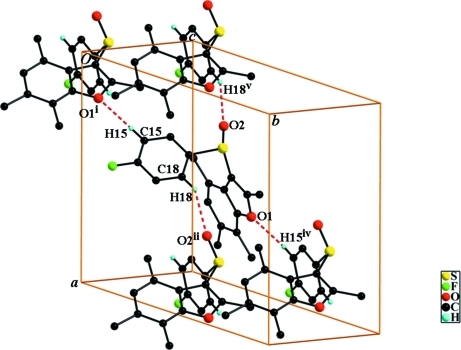
C—H···O interactions (dotted lines) in the crystal structure of the title compound. [Symmetry codes: (i) x - 1/2, - y + 1/2, z - 1; (ii) x + 1/2 , - y + 1/2, z; (iv) x + 1/2, - y + 1/2, z + 1; (v) x - 1/2, - y + 1/2, z.]

**Fig. 3.**
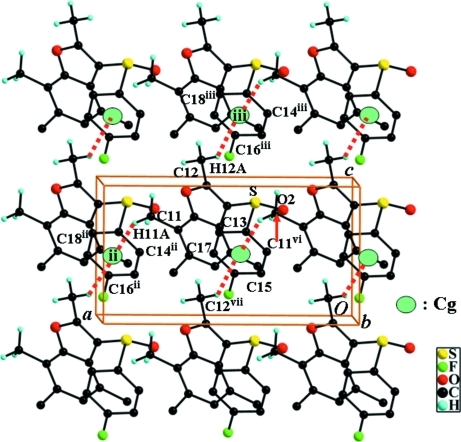
C—H···π interactions (dotted lines) in the crystal structure of the title compound. Cg denotes the ring centroid. [Symmetry codes: (ii) x + 1/2, - y + 1/2, z; (iii) x, y, z + 1; (vi) x - 1/2, - y + 1/2, z; (vii) x, y, z - 1.]

## supplementary crystallographic information

### Comment

Molecules containing benzofuran skeleton display significant biological activities such as antifungal (Aslam *et al.*, 2006), antitumor and antiviral (Galal *et al.*, 2009), antimicrobial (Khan *et al.*, 2005) properties. These compounds are widely occurring in nature (Akgul & Anil, 2003; Soekamto *et al.*, 2003). As a part of our ongoing studies of the effect of side chain substituents on the solid state structures of 2-methyl-3-phenylsulfinyl-1-benzofuran analogues (Choi *et al.*, 2007, 2008*a,b*), we report the crystal structure of the title compound (Fig. 1).

The benzofuran unit is essentially planar, with a mean deviation of 0.008 (2) Å from the least-squares plane defined by the nine constituent atoms. The 4-fluorophenyl ring is almost perpendicular to the plane of the benzofuran fragment [88.07 (5)°] and is tilted slightly towards it. The crystal packing (Fig. 2) is stabilized by two intermolecular C—H···O hydrogen bonds; the first between the 4-fluorophenyl H atom and the furan O atom, with a C15—H15···O1^i^, and the second between the 4-fluorophenyl H atom and the oxygen of the S═O unit, with a C18—H18···O2^ii^, respectively (Table 1).

The title compound is crystallized in the non-centrosymmetric space group *Pna*2_1_ in spite of having no asymmetric C atoms. The space group is caused by a right hand pseudo-helix along the *a* axis (Fig.3).

The molecular packing (Fig. 3) is further stabilized by two intermolecular C—H···π interactions; the first between the methyl H atom and the 4-fluorophenyl ring, with a C11—H11A···Cg^ii^, the second between the methyl H atom and 4-fluorophenyl ring of an adjacent molecule, with a C12—H12A···Cg^iii^, respectively (Table 1; Cg is the centroid of the C13–C18 4-fluorophenyl ring.)

### Experimental

77% 3-Chloroperoxybenzoic acid (291 mg, 1.3 mmol) was added in small portions to a stirred solution of 3-(4-fluorophenylsulfanyl)-2,4,6,7-tetramethyl-1-benzofuran (360 mg, 1.2 mmol) in dichloromethane (30 mL) at 273 K. After being stirred at room temperature for 3h, the mixture was washed with saturated sodium bicarbonate solution and the organic layer was separated, dried over magnesium sulfate, filtered and concentrated in vacuum. The residue was purified by column chromatography (hexane–ethyl acetate, 1:1 v/v) to afford the title compound as a colorless solid [yield 82%, m.p. 437–438 K; *R*_f_ = 0.53 (hexane–ethyl acetate, 1:1 v/v)]. Single crystals suitable for X-ray diffraction were prepared by slow evaporation of a solution of the title compound in ethyl acetate at room temperature.

### Refinement

All H atoms were positioned geometrically and refined using a riding model, with C—H = 0.95 Å for aryl and 0.98 Å for methyl H atoms. *U*_iso_(H) = 1.2*U*_eq_ (C) for aryl and 1.5*U*_eq_(C) for methyl H atoms. The reported Flack parameter was obtained by TWIN/BASF procedure in SHELXL (Sheldrick, 2008).

### Crystal data

**Table:**

C_18_H_17_FO_2_S	*F*(000) = 664
*M**_r_* = 316.38	*D*_x_ = 1.366 Mg m^−^^3^
Orthorhombic, *P**n**a*2_1_	Mo *K*α radiation, λ = 0.71073 Å
Hall symbol: P 2c -2n	Cell parameters from 4518 reflections
*a* = 12.0034 (5) Å	θ = 2.7–27.4°
*b* = 19.7455 (7) Å	µ = 0.23 mm^−^^1^
*c* = 6.4918 (3) Å	*T* = 169 K
*V* = 1538.64 (11) Å^3^	Block, colourless
*Z* = 4	0.18 × 0.17 × 0.16 mm

### Data collection

**Table:**

Bruker SMART APEXII CCD diffractometer	2280 independent reflections
Radiation source: Rotating Anode	2175 reflections with *I* > 2σ(*I*)
Bruker HELIOS graded multilayer optics	*R*_int_ = 0.024
Detector resolution: 10.0 pixels mm^-1^	θ_max_ = 27.5°, θ_min_ = 2.0°
φ and ω scans	*h* = −13→15
Absorption correction: multi-scan (*SADABS*; Bruker, 2009)	*k* = −25→25
*T*_min_ = 0.644, *T*_max_ = 0.746	*l* = −4→8
8107 measured reflections	

### Refinement

**Table:**

Refinement on *F*^2^	Secondary atom site location: difference Fourier map
Least-squares matrix: full	Hydrogen site location: difference Fourier map
*R*[*F*^2^ > 2σ(*F*^2^)] = 0.032	H-atom parameters constrained
*wR*(*F*^2^) = 0.082	*w* = 1/[σ^2^(*F*_o_^2^) + (0.0551*P*)^2^ + 0.1527*P*] where *P* = (*F*_o_^2^ + 2*F*_c_^2^)/3
*S* = 1.06	(Δ/σ)_max_ < 0.001
2280 reflections	Δρ_max_ = 0.25 e Å^−^^3^
204 parameters	Δρ_min_ = −0.28 e Å^−^^3^
1 restraint	Absolute structure: Flack (1983), 366 Friedel pairs
Primary atom site location: structure-invariant direct methods	Flack parameter: 0.01 (12)

### Special details

**Table:**

Geometry. All esds (except the esd in the dihedral angle between two l.s. planes) are estimated using the full covariance matrix. The cell esds are taken into account individually in the estimation of esds in distances, angles and torsion angles; correlations between esds in cell parameters are only used when they are defined by crystal symmetry. An approximate (isotropic) treatment of cell esds is used for estimating esds involving l.s. planes.
Refinement. Refinement of F^2^ against ALL reflections. The weighted R-factor wR and goodness of fit S are based on F^2^, conventional R-factors R are based on F, with F set to zero for negative F^2^. The threshold expression of F^2^ > 2sigma(F^2^) is used only for calculating R-factors(gt) etc. and is not relevant to the choice of reflections for refinement. R-factors based on F^2^ are statistically about twice as large as those based on F, and R- factors based on ALL data will be even larger.

### Fractional atomic coordinates and isotropic or equivalent isotropic displacement parameters (Å^2^)

**Table:**

	*x*	*y*	*z*	*U*_iso_*/*U*_eq_	
S	0.38790 (4)	0.25971 (2)	0.83129 (12)	0.02606 (12)	
F	0.48409 (13)	0.07177 (8)	0.1553 (3)	0.0599 (5)	
O1	0.66099 (10)	0.36300 (7)	0.9163 (2)	0.0258 (3)	
O2	0.28018 (11)	0.29365 (7)	0.7817 (3)	0.0352 (4)	
C1	0.49909 (14)	0.31749 (9)	0.8113 (3)	0.0235 (4)	
C2	0.52977 (15)	0.36899 (9)	0.6611 (3)	0.0232 (4)	
C3	0.48697 (16)	0.39560 (10)	0.4772 (3)	0.0273 (4)	
C4	0.54918 (17)	0.44634 (9)	0.3828 (3)	0.0307 (4)	
H4	0.5214	0.4655	0.2587	0.037*	
C5	0.65120 (17)	0.47098 (9)	0.4609 (4)	0.0306 (4)	
C6	0.69347 (16)	0.44561 (9)	0.6454 (4)	0.0265 (4)	
C7	0.62992 (15)	0.39505 (9)	0.7356 (3)	0.0241 (4)	
C8	0.57968 (16)	0.31611 (9)	0.9575 (3)	0.0246 (4)	
C9	0.38031 (17)	0.37126 (12)	0.3799 (3)	0.0315 (5)	
H9A	0.3537	0.4051	0.2812	0.047*	
H9B	0.3239	0.3644	0.4871	0.047*	
H9C	0.3939	0.3284	0.3081	0.047*	
C10	0.71324 (19)	0.52419 (10)	0.3411 (5)	0.0419 (5)	
H10A	0.7075	0.5678	0.4126	0.063*	
H10B	0.6809	0.5282	0.2031	0.063*	
H10C	0.7918	0.5112	0.3297	0.063*	
C11	0.80130 (18)	0.46820 (11)	0.7418 (4)	0.0362 (5)	
H11A	0.8622	0.4395	0.6920	0.054*	
H11B	0.7959	0.4643	0.8920	0.054*	
H11C	0.8160	0.5154	0.7043	0.054*	
C12	0.59977 (17)	0.27324 (11)	1.1404 (4)	0.0297 (4)	
H12A	0.5354	0.2436	1.1630	0.045*	
H12B	0.6109	0.3021	1.2615	0.045*	
H12C	0.6664	0.2455	1.1179	0.045*	
C13	0.42124 (15)	0.20619 (9)	0.6169 (3)	0.0248 (4)	
C14	0.33994 (16)	0.19268 (9)	0.4725 (4)	0.0274 (4)	
H14	0.2697	0.2146	0.4812	0.033*	
C15	0.36070 (16)	0.14697 (9)	0.3142 (4)	0.0311 (4)	
H15	0.3059	0.1375	0.2127	0.037*	
C16	0.46320 (18)	0.11590 (10)	0.3093 (4)	0.0370 (5)	
C17	0.54509 (18)	0.12796 (11)	0.4537 (4)	0.0387 (5)	
H17	0.6145	0.1050	0.4460	0.046*	
C18	0.52465 (16)	0.17380 (10)	0.6092 (4)	0.0309 (4)	
H18	0.5800	0.1832	0.7097	0.037*	

### Atomic displacement parameters (Å^2^)

**Table:**

	*U*^11^	*U*^22^	*U*^33^	*U*^12^	*U*^13^	*U*^23^
S	0.0189 (2)	0.0315 (2)	0.0278 (2)	−0.00380 (16)	0.0001 (2)	0.0014 (2)
F	0.0513 (8)	0.0624 (9)	0.0659 (11)	0.0177 (7)	−0.0131 (9)	−0.0339 (9)
O1	0.0215 (7)	0.0287 (6)	0.0273 (7)	−0.0043 (5)	−0.0026 (6)	0.0005 (6)
O2	0.0195 (7)	0.0421 (8)	0.0440 (10)	0.0022 (6)	0.0003 (6)	−0.0035 (7)
C1	0.0197 (8)	0.0252 (8)	0.0255 (9)	−0.0019 (6)	0.0007 (8)	−0.0010 (8)
C2	0.0212 (8)	0.0233 (8)	0.0252 (9)	0.0028 (7)	0.0013 (7)	−0.0002 (8)
C3	0.0268 (9)	0.0274 (9)	0.0276 (10)	0.0066 (7)	−0.0018 (8)	−0.0002 (8)
C4	0.0328 (10)	0.0297 (9)	0.0294 (11)	0.0072 (8)	0.0002 (8)	0.0044 (8)
C5	0.0326 (10)	0.0221 (8)	0.0372 (11)	0.0048 (8)	0.0075 (10)	0.0043 (9)
C6	0.0257 (9)	0.0216 (8)	0.0322 (10)	0.0009 (7)	0.0042 (9)	−0.0023 (8)
C7	0.0223 (9)	0.0241 (8)	0.0258 (9)	0.0019 (6)	0.0010 (8)	−0.0015 (8)
C8	0.0211 (9)	0.0263 (9)	0.0263 (9)	−0.0023 (7)	0.0005 (8)	−0.0026 (7)
C9	0.0289 (10)	0.0375 (10)	0.0282 (12)	0.0049 (8)	−0.0065 (8)	0.0007 (8)
C10	0.0422 (11)	0.0345 (10)	0.0490 (13)	0.0003 (9)	0.0082 (13)	0.0131 (12)
C11	0.0327 (11)	0.0328 (10)	0.0431 (12)	−0.0084 (8)	0.0013 (10)	−0.0025 (10)
C12	0.0259 (10)	0.0351 (10)	0.0279 (10)	−0.0026 (8)	−0.0022 (9)	0.0053 (8)
C13	0.0205 (9)	0.0235 (8)	0.0303 (9)	−0.0031 (7)	−0.0028 (8)	0.0019 (8)
C14	0.0212 (9)	0.0268 (8)	0.0342 (10)	−0.0014 (7)	−0.0054 (8)	0.0023 (8)
C15	0.0275 (9)	0.0294 (9)	0.0364 (12)	−0.0023 (7)	−0.0083 (10)	−0.0017 (10)
C16	0.0374 (11)	0.0314 (9)	0.0421 (13)	0.0042 (8)	−0.0050 (11)	−0.0079 (10)
C17	0.0277 (11)	0.0358 (11)	0.0525 (14)	0.0074 (8)	−0.0074 (11)	−0.0071 (11)
C18	0.0228 (9)	0.0297 (9)	0.0403 (11)	0.0010 (8)	−0.0071 (9)	0.0002 (9)

### Geometric parameters (Å, °)

**Table:**

S—O2	1.4915 (14)	C9—H9C	0.9800
S—C1	1.7606 (18)	C10—H10A	0.9800
S—C13	1.793 (2)	C10—H10B	0.9800
F—C16	1.350 (3)	C10—H10C	0.9800
O1—C8	1.372 (2)	C11—H11A	0.9800
O1—C7	1.384 (2)	C11—H11B	0.9800
C1—C8	1.356 (3)	C11—H11C	0.9800
C1—C2	1.456 (3)	C12—H12A	0.9800
C2—C7	1.394 (3)	C12—H12B	0.9800
C2—C3	1.402 (3)	C12—H12C	0.9800
C3—C4	1.392 (3)	C13—C14	1.379 (3)
C3—C9	1.506 (3)	C13—C18	1.397 (3)
C4—C5	1.412 (3)	C14—C15	1.390 (3)
C4—H4	0.9500	C14—H14	0.9500
C5—C6	1.394 (3)	C15—C16	1.375 (3)
C5—C10	1.504 (3)	C15—H15	0.9500
C6—C7	1.386 (3)	C16—C17	1.379 (3)
C6—C11	1.505 (3)	C17—C18	1.378 (3)
C8—C12	1.478 (3)	C17—H17	0.9500
C9—H9A	0.9800	C18—H18	0.9500
C9—H9B	0.9800		
			
O2—S—C1	110.49 (9)	C5—C10—H10B	109.5
O2—S—C13	106.91 (10)	H10A—C10—H10B	109.5
C1—S—C13	98.95 (9)	C5—C10—H10C	109.5
C8—O1—C7	106.41 (15)	H10A—C10—H10C	109.5
C8—C1—C2	107.61 (16)	H10B—C10—H10C	109.5
C8—C1—S	118.44 (15)	C6—C11—H11A	109.5
C2—C1—S	133.91 (15)	C6—C11—H11B	109.5
C7—C2—C3	118.25 (18)	H11A—C11—H11B	109.5
C7—C2—C1	104.08 (16)	C6—C11—H11C	109.5
C3—C2—C1	137.67 (18)	H11A—C11—H11C	109.5
C4—C3—C2	116.62 (18)	H11B—C11—H11C	109.5
C4—C3—C9	120.07 (19)	C8—C12—H12A	109.5
C2—C3—C9	123.30 (18)	C8—C12—H12B	109.5
C3—C4—C5	123.73 (19)	H12A—C12—H12B	109.5
C3—C4—H4	118.1	C8—C12—H12C	109.5
C5—C4—H4	118.1	H12A—C12—H12C	109.5
C6—C5—C4	120.03 (18)	H12B—C12—H12C	109.5
C6—C5—C10	121.0 (2)	C14—C13—C18	121.06 (19)
C4—C5—C10	119.0 (2)	C14—C13—S	118.92 (15)
C7—C6—C5	114.95 (18)	C18—C13—S	119.72 (16)
C7—C6—C11	120.7 (2)	C13—C14—C15	120.07 (18)
C5—C6—C11	124.32 (19)	C13—C14—H14	120.0
O1—C7—C6	122.64 (18)	C15—C14—H14	120.0
O1—C7—C2	110.96 (16)	C16—C15—C14	117.8 (2)
C6—C7—C2	126.40 (19)	C16—C15—H15	121.1
C1—C8—O1	110.94 (17)	C14—C15—H15	121.1
C1—C8—C12	133.67 (17)	F—C16—C15	118.1 (2)
O1—C8—C12	115.30 (17)	F—C16—C17	118.86 (19)
C3—C9—H9A	109.5	C15—C16—C17	123.0 (2)
C3—C9—H9B	109.5	C18—C17—C16	118.98 (19)
H9A—C9—H9B	109.5	C18—C17—H17	120.5
C3—C9—H9C	109.5	C16—C17—H17	120.5
H9A—C9—H9C	109.5	C17—C18—C13	119.01 (19)
H9B—C9—H9C	109.5	C17—C18—H18	120.5
C5—C10—H10A	109.5	C13—C18—H18	120.5
			
O2—S—C1—C8	137.99 (16)	C11—C6—C7—C2	−179.11 (19)
C13—S—C1—C8	−110.10 (17)	C3—C2—C7—O1	−179.38 (16)
O2—S—C1—C2	−44.4 (2)	C1—C2—C7—O1	0.3 (2)
C13—S—C1—C2	67.5 (2)	C3—C2—C7—C6	−0.1 (3)
C8—C1—C2—C7	−0.4 (2)	C1—C2—C7—C6	179.60 (18)
S—C1—C2—C7	−178.16 (16)	C2—C1—C8—O1	0.3 (2)
C8—C1—C2—C3	179.2 (2)	S—C1—C8—O1	178.51 (13)
S—C1—C2—C3	1.4 (4)	C2—C1—C8—C12	−176.0 (2)
C7—C2—C3—C4	0.3 (3)	S—C1—C8—C12	2.2 (3)
C1—C2—C3—C4	−179.3 (2)	C7—O1—C8—C1	−0.1 (2)
C7—C2—C3—C9	179.36 (18)	C7—O1—C8—C12	176.90 (17)
C1—C2—C3—C9	−0.2 (4)	O2—S—C13—C14	−13.62 (19)
C2—C3—C4—C5	0.6 (3)	C1—S—C13—C14	−128.35 (17)
C9—C3—C4—C5	−178.50 (19)	O2—S—C13—C18	172.58 (16)
C3—C4—C5—C6	−1.7 (3)	C1—S—C13—C18	57.85 (18)
C3—C4—C5—C10	177.73 (19)	C18—C13—C14—C15	−1.0 (3)
C4—C5—C6—C7	1.7 (3)	S—C13—C14—C15	−174.70 (16)
C10—C5—C6—C7	−177.71 (18)	C13—C14—C15—C16	0.7 (3)
C4—C5—C6—C11	179.86 (19)	C14—C15—C16—F	−179.6 (2)
C10—C5—C6—C11	0.4 (3)	C14—C15—C16—C17	0.2 (4)
C8—O1—C7—C6	−179.43 (18)	F—C16—C17—C18	179.0 (2)
C8—O1—C7—C2	−0.12 (19)	C15—C16—C17—C18	−0.8 (4)
C5—C6—C7—O1	178.31 (17)	C16—C17—C18—C13	0.6 (4)
C11—C6—C7—O1	0.1 (3)	C14—C13—C18—C17	0.3 (3)
C5—C6—C7—C2	−0.9 (3)	S—C13—C18—C17	173.99 (18)

### Hydrogen-bond geometry (Å, °)

**Table:**

Cg is the centroid of the C13–C18 ring.

**Table:**

*D*—H···*A*	*D*—H	H···*A*	*D*···*A*	*D*—H···*A*
C15—H15···O1^i^	0.95	2.59	3.529 (3)	169
C18—H18···O2^ii^	0.95	2.49	3.328 (2)	147
C11—H11A···Cg^ii^	0.98	2.66	3.553 (3)	152
C12—H12A···Cg^iii^	0.98	2.78	3.590 (3)	141

Symmetry codes: (i) *x*−1/2, −*y*+1/2, *z*−1; (ii) *x*+1/2, −*y*+1/2, *z*; (iii) *x*, *y*, *z*+1.
